# Di­aqua­bis­(2-ethyl-5-methyl­imidazole-4-sulfonato-κ^2^ *N* ^3^,*O*)nickel(II) dihydrate

**DOI:** 10.1107/S1600536813032169

**Published:** 2013-12-14

**Authors:** Andrew P. Purdy, Ray J. Butcher

**Affiliations:** a Chemistry Division, Code 6100 Naval Research Laboratory, 4555 Overlook Avenue SW, Washington, DC 20375, USA; b Department of Chemistry, Howard University, 525 College Street NW, Washington, DC 20059, USA

## Abstract

In the title complex, [Ni(C_6_H_9_N_2_O_3_S)_2_(H_2_O)_2_]·2H_2_O, the Ni^II^ atom lies on an inversion center and is chelated by N and O atoms of two symmetry-equivalent imidazole­sulfonate ligands in the basal plane, and two water O atoms in axial positions in an overall octa­hedral configuration. The crystal structure displays O—H⋯O and N—H⋯O hydrogen bonds, which connect the components into an extended three-dimensional network.

## Related literature   

For examples of Ni–sulfonate complexes and MOFs, see: Lobana *et al.* (2004 ▶); Forbes & Sevov (2009 ▶); Kim *et al.* (2004 ▶); Yang *et al.* (2010 ▶). A small number of structurally characterized imidazole sulfonates are known, see: Kuhn *et al.* (2001 ▶, 2002 ▶); Chidambaram *et al.* (1988 ▶). The 2-ethyl-4-methyl-5-sulfonate ligand is described by Purdy *et al.* (2007 ▶) and Purdy & Butcher (2011 ▶).
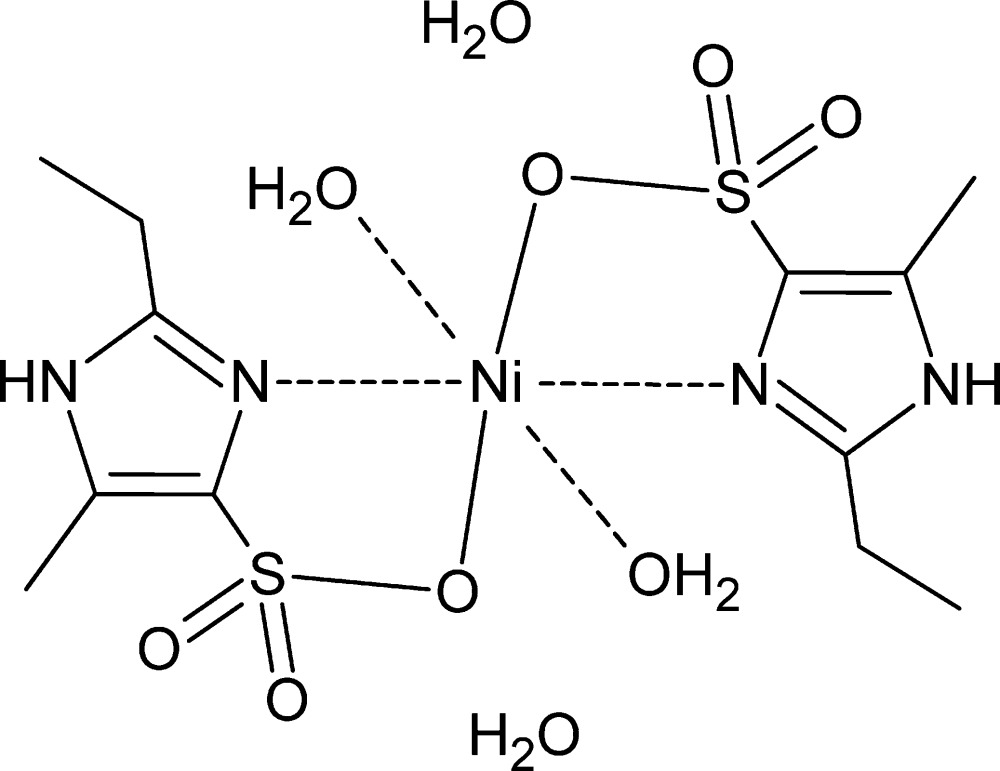


## Experimental   

### 

#### Crystal data  

[Ni(C_6_H_9_N_2_O_3_S)_2_(H_2_O)_2_]·2H_2_O
*M* *_r_* = 509.20
Monoclinic, 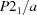

*a* = 7.6037 (2) Å
*b* = 16.8934 (4) Å
*c* = 8.6574 (3) Å
β = 111.303 (3)°
*V* = 1036.08 (5) Å^3^
*Z* = 2
Cu *K*α radiation
μ = 3.77 mm^−1^
*T* = 123 K
0.52 × 0.46 × 0.35 mm

#### Data collection  

Agilent Xcalibur Ruby Gemini diffractometer
Absorption correction: multi-scan (*CrysAlis PRO*; Agilent, 2012 ▶) *T*_min_ = 0.209, *T*_max_ = 1.000
2126 measured reflections
2126 independent reflections
2062 reflections with *I* > 2σ(*I*)

#### Refinement  

*R*[*F* ^2^ > 2σ(*F* ^2^)] = 0.045
*wR*(*F* ^2^) = 0.112
*S* = 1.18
2126 reflections
151 parameters
48 restraints
H atoms treated by a mixture of independent and constrained refinement
Δρ_max_ = 0.73 e Å^−3^
Δρ_min_ = −0.54 e Å^−3^

### 

Data collection: *CrysAlis PRO* (Agilent, 2012 ▶); cell refinement: *CrysAlis PRO*; data reduction: *CrysAlis PRO*; program(s) used to solve structure: *SHELXS97* (Sheldrick, 2008 ▶); program(s) used to refine structure: *SHELXL97* (Sheldrick, 2008 ▶); molecular graphics: *SHELXTL* (Sheldrick, 2008 ▶); software used to prepare material for publication: *SHELXTL*.

## Supplementary Material

Crystal structure: contains datablock(s) I, New_Global_Publ_Block. DOI: 10.1107/S1600536813032169/jj2177sup1.cif

Structure factors: contains datablock(s) I. DOI: 10.1107/S1600536813032169/jj2177Isup2.hkl

Additional supporting information:  crystallographic information; 3D view; checkCIF report

## Figures and Tables

**Table 1:** Hydrogen-bond geometry (Å, °)

*D*—H⋯*A*	*D*—H	H⋯*A*	*D*⋯*A*	*D*—H⋯*A*
O1*W*—H1*W*1⋯O2*W*	0.82 (2)	2.02 (2)	2.837 (3)	174 (4)
O1*W*—H1*W*2⋯O3^i^	0.81 (2)	1.96 (2)	2.739 (3)	161 (4)
O2*W*—H2*W*1⋯O2^ii^	0.80 (2)	1.97 (2)	2.751 (3)	167 (4)
O2*W*—H2*W*2⋯O2^iii^	0.80 (2)	1.94 (2)	2.723 (3)	169 (4)
N2—H2*A*⋯O2*W* ^iv^	0.88	1.94	2.818 (3)	174

Symmetry codes: (i) 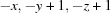; (ii) 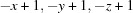; (iii) 
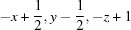
; (iv) 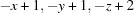.

**Fig. 1.**
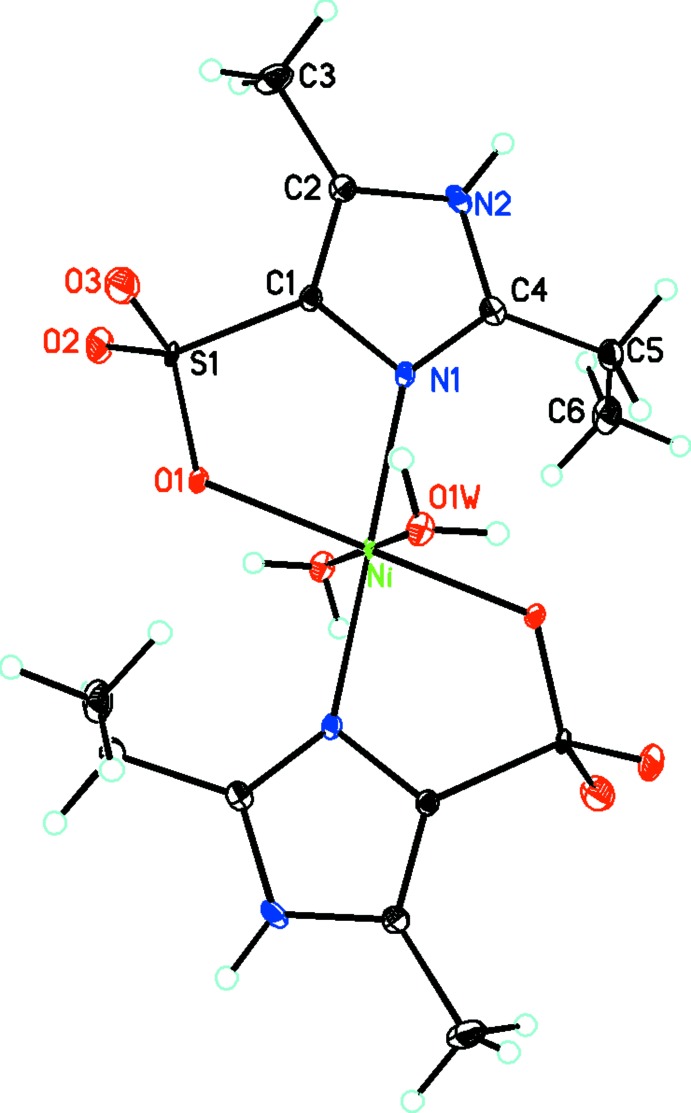
Diagram of C12H22N4O8S2Ni, with uncoordinated water molecules not shown.

**Fig. 2.**
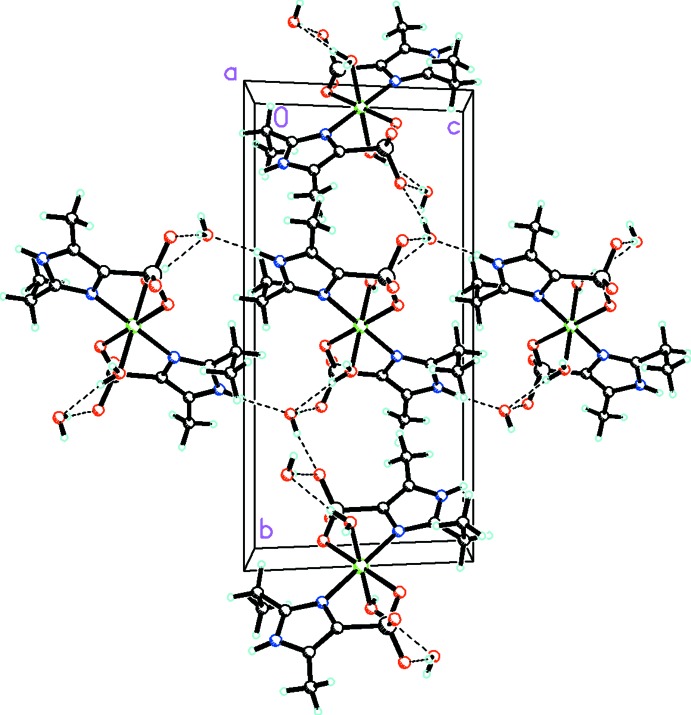
Packing diagram viewed down the c axis, displaying the hydrogen bonded interactions of both the coordinated and uncoordinated water molecules.

## supplementary crystallographic information

### 1. Comment

The Ni atom lies on an inversion center and is chelated by N1 and O1 of the 2 symmetry equivalent imidazolesulfonate ligands in a plane, and 2 axial water molecules coordinated in an overall octahedral configuration. All of the Ni—O bond lengths (2.083 (2), 2.094 (2) Å) are about the same, and are in the normal range for octahedral coordinated Ni (2.05–2.12 Å). The largest deviation from the 90 ° angles of the octahedron is N1—Ni—O1, a result of the sulfonate group being attached to the imidazole ring. Two additional uncoordinated water molecules are present, but do not lie on any symmetry elements. There are no links other than hydrogen bonds between molecules, in contrast to the analogous unsolvated Cu(II) derivative (Purdy & Butcher, 2011). Hydrogen bonding links all the water molecules and N2, O2, and O3 into an extended structure in all three dimensions.

### 2. Experimental

A solution of the potassium salt of the 2-ethyl-4-methyl-imidazole-5-sulfonic acid was prepared by combining 1 g (5.25 mmol) of the free acid with 1.5 equivalents of KOH solution, and diluting the solution to 1*M* based on K^+^. (All solutions were made with distilled water.) Two test reactions were done in vials with 0.5 *M* solutions of Ni(BF_4_)_2_·6H_2_O and MnSO_4_·H_2_O, a 0.2 ml metered pipet was used for the additions, and each vial contained one addition of all three solutions. After 1 month, one vial were heated to a boil and allowed to cool and the other remained at room temperature. After one year, large blue crystals of the title compound grew in the solution that was not heated.

### Crystal data

**Table:**

[Ni(C_6_H_9_N_2_O_3_S)_2_(H_2_O)_2_]·2H_2_O	*F*(000) = 532
*M**_r_* = 509.20	*D*_x_ = 1.632 Mg m^−^^3^
Monoclinic, *P*2_1_/*a*	Cu *K*α radiation, λ = 1.54178 Å
Hall symbol: -P 2yab	Cell parameters from 4614 reflections
*a* = 7.6037 (2) Å	θ = 5.2–75.5°
*b* = 16.8934 (4) Å	µ = 3.77 mm^−^^1^
*c* = 8.6574 (3) Å	*T* = 123 K
β = 111.303 (3)°	Block, blue
*V* = 1036.08 (5) Å^3^	0.52 × 0.46 × 0.35 mm
*Z* = 2	

### Data collection

**Table:**

Agilent Xcalibur Ruby Gemini diffractometer	2126 independent reflections
Radiation source: Enhance (Cu) X-ray Source	2062 reflections with *I* > 2σ(*I*)
Graphite monochromator	*R*_int_ = 0.000
Detector resolution: 10.5081 pixels mm^-1^	θ_max_ = 75.8°, θ_min_ = 5.2°
ω scans	*h* = −9→8
Absorption correction: multi-scan (*CrysAlis PRO*; Agilent, 2012)	*k* = 0→21
*T*_min_ = 0.209, *T*_max_ = 1.000	*l* = 0→10
2126 measured reflections	

### Refinement

**Table:**

Refinement on *F*^2^	Primary atom site location: structure-invariant direct methods
Least-squares matrix: full	Secondary atom site location: difference Fourier map
*R*[*F*^2^ > 2σ(*F*^2^)] = 0.045	Hydrogen site location: inferred from neighbouring sites
*wR*(*F*^2^) = 0.112	H atoms treated by a mixture of independent and constrained refinement
*S* = 1.18	*w* = 1/[σ^2^(*F*_o_^2^) + (0.0411*P*)^2^ + 2.2827*P*] where *P* = (*F*_o_^2^ + 2*F*_c_^2^)/3
2126 reflections	(Δ/σ)_max_ < 0.001
151 parameters	Δρ_max_ = 0.73 e Å^−^^3^
48 restraints	Δρ_min_ = −0.54 e Å^−^^3^

### Special details

**Table:**

Geometry. All e.s.d.'s (except the e.s.d. in the dihedral angle between two l.s. planes) are estimated using the full covariance matrix. The cell e.s.d.'s are taken into account individually in the estimation of e.s.d.'s in distances, angles and torsion angles; correlations between e.s.d.'s in cell parameters are only used when they are defined by crystal symmetry. An approximate (isotropic) treatment of cell e.s.d.'s is used for estimating e.s.d.'s involving l.s. planes.
Refinement. Refinement of *F*^2^ against ALL reflections. The weighted *R*-factor *wR* and goodness of fit *S* are based on *F*^2^, conventional *R*-factors *R* are based on *F*, with *F* set to zero for negative *F*^2^. The threshold expression of *F*^2^ > σ(*F*^2^) is used only for calculating *R*-factors(gt) *etc*. and is not relevant to the choice of reflections for refinement. *R*-factors based on *F*^2^ are statistically about twice as large as those based on *F*, and *R*- factors based on ALL data will be even larger.

### Fractional atomic coordinates and isotropic or equivalent isotropic displacement parameters (Å^2^)

**Table:**

	*x*	*y*	*z*	*U*_iso_*/*U*_eq_	
Ni	0.5000	0.5000	0.5000	0.00455 (19)	
S1	0.13784 (8)	0.60394 (4)	0.38692 (7)	0.00676 (18)	
O1	0.2439 (3)	0.54424 (11)	0.3337 (2)	0.0082 (4)	
O2	0.1485 (3)	0.68145 (12)	0.3149 (2)	0.0148 (4)	
O3	−0.0536 (3)	0.58051 (13)	0.3598 (2)	0.0165 (4)	
O1W	0.3581 (3)	0.40250 (11)	0.5452 (2)	0.0104 (4)	
H1W1	0.422 (5)	0.378 (2)	0.629 (4)	0.027 (11)*	
H1W2	0.267 (4)	0.417 (2)	0.563 (5)	0.027 (11)*	
O2W	0.5754 (3)	0.30842 (12)	0.8196 (2)	0.0129 (4)	
H2W1	0.667 (4)	0.309 (2)	0.795 (5)	0.014 (9)*	
H2W2	0.519 (5)	0.2680 (16)	0.790 (5)	0.019 (10)*	
N1	0.4281 (3)	0.56704 (13)	0.6667 (3)	0.0065 (4)	
N2	0.3814 (3)	0.63709 (13)	0.8610 (3)	0.0094 (5)	
H2A	0.3996	0.6567	0.9598	0.011*	
C1	0.2651 (4)	0.61089 (15)	0.5997 (3)	0.0072 (5)	
C2	0.2329 (4)	0.65519 (15)	0.7178 (3)	0.0085 (5)	
C3	0.0834 (4)	0.71410 (17)	0.7081 (4)	0.0155 (6)	
H3A	−0.0366	0.6972	0.6241	0.023*	
H3B	0.1188	0.7659	0.6777	0.023*	
H3C	0.0696	0.7179	0.8161	0.023*	
C4	0.4953 (4)	0.58422 (15)	0.8257 (3)	0.0083 (5)	
C5	0.6769 (4)	0.55417 (16)	0.9505 (3)	0.0110 (5)	
H5A	0.6884	0.4968	0.9328	0.013*	
H5B	0.6752	0.5615	1.0634	0.013*	
C6	0.8474 (4)	0.59737 (17)	0.9371 (4)	0.0152 (6)	
H6A	0.9637	0.5738	1.0147	0.023*	
H6B	0.8420	0.6534	0.9641	0.023*	
H6C	0.8457	0.5925	0.8237	0.023*	

### Atomic displacement parameters (Å^2^)

**Table:**

	*U*^11^	*U*^22^	*U*^33^	*U*^12^	*U*^13^	*U*^23^
Ni	0.0030 (3)	0.0058 (3)	0.0026 (3)	0.0012 (2)	−0.0017 (2)	−0.0004 (2)
S1	0.0039 (3)	0.0098 (3)	0.0042 (3)	0.0027 (2)	−0.0014 (2)	0.0007 (2)
O1	0.0067 (7)	0.0098 (7)	0.0051 (7)	0.0030 (6)	−0.0012 (5)	−0.0014 (6)
O2	0.0208 (11)	0.0099 (9)	0.0122 (9)	0.0059 (8)	0.0043 (8)	0.0056 (7)
O3	0.0112 (8)	0.0241 (8)	0.0131 (7)	−0.0003 (6)	0.0031 (6)	−0.0012 (6)
O1W	0.0087 (9)	0.0105 (9)	0.0108 (9)	0.0002 (7)	0.0023 (7)	0.0014 (7)
O2W	0.0164 (10)	0.0113 (9)	0.0105 (9)	−0.0051 (8)	0.0042 (8)	−0.0044 (7)
N1	0.0054 (8)	0.0070 (8)	0.0056 (7)	0.0000 (6)	0.0002 (6)	0.0002 (6)
N2	0.0121 (11)	0.0102 (11)	0.0050 (10)	−0.0014 (8)	0.0020 (8)	−0.0025 (8)
C1	0.0065 (8)	0.0075 (8)	0.0065 (8)	−0.0002 (7)	0.0008 (7)	−0.0005 (7)
C2	0.0078 (12)	0.0081 (11)	0.0085 (12)	−0.0012 (9)	0.0015 (10)	−0.0021 (9)
C3	0.0132 (14)	0.0129 (13)	0.0197 (14)	0.0028 (11)	0.0052 (11)	−0.0049 (11)
C4	0.0110 (13)	0.0059 (11)	0.0077 (12)	−0.0021 (9)	0.0029 (10)	−0.0003 (9)
C5	0.0115 (9)	0.0116 (9)	0.0081 (8)	0.0007 (7)	0.0013 (7)	0.0007 (7)
C6	0.0104 (13)	0.0177 (14)	0.0122 (13)	−0.0012 (10)	−0.0023 (10)	−0.0014 (10)

### Geometric parameters (Å, º)

**Table:**

Ni—N1	2.058 (2)	N2—C4	1.354 (4)
Ni—N1^i^	2.058 (2)	N2—C2	1.374 (3)
Ni—O1W	2.0825 (19)	N2—H2A	0.8800
Ni—O1W^i^	2.0825 (19)	C1—C2	1.359 (4)
Ni—O1	2.0941 (18)	C2—C3	1.490 (4)
Ni—O1^i^	2.0941 (18)	C3—H3A	0.9800
S1—O3	1.443 (2)	C3—H3B	0.9800
S1—O2	1.465 (2)	C3—H3C	0.9800
S1—O1	1.4662 (18)	C4—C5	1.499 (4)
S1—C1	1.746 (3)	C5—C6	1.528 (4)
O1W—H1W1	0.823 (19)	C5—H5A	0.9900
O1W—H1W2	0.808 (19)	C5—H5B	0.9900
O2W—H2W1	0.798 (18)	C6—H6A	0.9800
O2W—H2W2	0.796 (18)	C6—H6B	0.9800
N1—C4	1.315 (3)	C6—H6C	0.9800
N1—C1	1.378 (3)		
			
N1—Ni—N1^i^	180.0	C4—N2—H2A	125.5
N1—Ni—O1W	90.90 (8)	C2—N2—H2A	125.5
N1^i^—Ni—O1W	89.10 (8)	C2—C1—N1	111.1 (2)
N1—Ni—O1W^i^	89.10 (8)	C2—C1—S1	130.4 (2)
N1^i^—Ni—O1W^i^	90.90 (8)	N1—C1—S1	118.48 (19)
O1W—Ni—O1W^i^	180.00 (6)	C1—C2—N2	104.1 (2)
N1—Ni—O1	82.42 (8)	C1—C2—C3	131.9 (2)
N1^i^—Ni—O1	97.58 (8)	N2—C2—C3	124.0 (2)
O1W—Ni—O1	89.75 (8)	C2—C3—H3A	109.5
O1W^i^—Ni—O1	90.25 (8)	C2—C3—H3B	109.5
N1—Ni—O1^i^	97.58 (8)	H3A—C3—H3B	109.5
N1^i^—Ni—O1^i^	82.42 (8)	C2—C3—H3C	109.5
O1W—Ni—O1^i^	90.25 (8)	H3A—C3—H3C	109.5
O1W^i^—Ni—O1^i^	89.75 (8)	H3B—C3—H3C	109.5
O1—Ni—O1^i^	180.00 (14)	N1—C4—N2	110.2 (2)
O3—S1—O2	112.61 (13)	N1—C4—C5	125.8 (2)
O3—S1—O1	113.45 (12)	N2—C4—C5	123.9 (2)
O2—S1—O1	111.06 (11)	C4—C5—C6	111.6 (2)
O3—S1—C1	109.24 (12)	C4—C5—H5A	109.3
O2—S1—C1	107.09 (12)	C6—C5—H5A	109.3
O1—S1—C1	102.73 (11)	C4—C5—H5B	109.3
S1—O1—Ni	120.66 (10)	C6—C5—H5B	109.3
Ni—O1W—H1W1	112 (3)	H5A—C5—H5B	108.0
Ni—O1W—H1W2	109 (3)	C5—C6—H6A	109.5
H1W1—O1W—H1W2	105 (3)	C5—C6—H6B	109.5
H2W1—O2W—H2W2	110 (3)	H6A—C6—H6B	109.5
C4—N1—C1	105.7 (2)	C5—C6—H6C	109.5
C4—N1—Ni	138.96 (19)	H6A—C6—H6C	109.5
C1—N1—Ni	115.34 (16)	H6B—C6—H6C	109.5
C4—N2—C2	109.0 (2)		
			
O3—S1—O1—Ni	−124.06 (13)	Ni—N1—C1—S1	1.0 (3)
O2—S1—O1—Ni	107.92 (14)	O3—S1—C1—C2	−57.4 (3)
C1—S1—O1—Ni	−6.27 (15)	O2—S1—C1—C2	64.8 (3)
N1—Ni—O1—S1	6.10 (13)	O1—S1—C1—C2	−178.1 (3)
N1^i^—Ni—O1—S1	−173.90 (13)	O3—S1—C1—N1	124.0 (2)
O1W—Ni—O1—S1	97.04 (13)	O2—S1—C1—N1	−113.8 (2)
O1W^i^—Ni—O1—S1	−82.96 (13)	O1—S1—C1—N1	3.2 (2)
O1^i^—Ni—O1—S1	89 (6)	N1—C1—C2—N2	−0.2 (3)
N1^i^—Ni—N1—C4	81 (8)	S1—C1—C2—N2	−178.9 (2)
O1W—Ni—N1—C4	89.7 (3)	N1—C1—C2—C3	177.0 (3)
O1W^i^—Ni—N1—C4	−90.3 (3)	S1—C1—C2—C3	−1.7 (5)
O1—Ni—N1—C4	179.4 (3)	C4—N2—C2—C1	0.2 (3)
O1^i^—Ni—N1—C4	−0.6 (3)	C4—N2—C2—C3	−177.3 (3)
N1^i^—Ni—N1—C1	−102 (8)	C1—N1—C4—N2	−0.1 (3)
O1W—Ni—N1—C1	−93.13 (18)	Ni—N1—C4—N2	177.21 (19)
O1W^i^—Ni—N1—C1	86.87 (18)	C1—N1—C4—C5	−176.8 (2)
O1—Ni—N1—C1	−3.50 (17)	Ni—N1—C4—C5	0.5 (4)
O1^i^—Ni—N1—C1	176.50 (17)	C2—N2—C4—N1	0.0 (3)
C4—N1—C1—C2	0.2 (3)	C2—N2—C4—C5	176.7 (2)
Ni—N1—C1—C2	−177.83 (17)	N1—C4—C5—C6	76.8 (3)
C4—N1—C1—S1	179.10 (18)	N2—C4—C5—C6	−99.4 (3)

Symmetry code: (i) −*x*+1, −*y*+1, −*z*+1.

### Hydrogen-bond geometry (Å, º)

**Table:**

*D*—H···*A*	*D*—H	H···*A*	*D*···*A*	*D*—H···*A*
O1*W*—H1*W*1···O2*W*	0.82 (2)	2.02 (2)	2.837 (3)	174 (4)
O1*W*—H1*W*2···O3^ii^	0.81 (2)	1.96 (2)	2.739 (3)	161 (4)
O2*W*—H2*W*1···O2^i^	0.80 (2)	1.97 (2)	2.751 (3)	167 (4)
O2*W*—H2*W*1···S1^i^	0.80 (2)	2.92 (3)	3.602 (2)	145 (3)
O2*W*—H2*W*2···O2^iii^	0.80 (2)	1.94 (2)	2.723 (3)	169 (4)
N2—H2*A*···O2*W*^iv^	0.88	1.94	2.818 (3)	174

Symmetry codes: (i) −*x*+1, −*y*+1, −*z*+1; (ii) −*x*, −*y*+1, −*z*+1; (iii) −*x*+1/2, *y*−1/2, −*z*+1; (iv) −*x*+1, −*y*+1, −*z*+2.
